# Molecular subtypes of colorectal cancer in the era of precision oncotherapy: Current inspirations and future challenges

**DOI:** 10.1002/cam4.70041

**Published:** 2024-07-26

**Authors:** Qin Dang, Lulu Zuo, Xinru Hu, Zhaokai Zhou, Shuang Chen, Shutong Liu, Yuhao Ba, Anning Zuo, Hui Xu, Siyuan Weng, Yuyuan Zhang, Peng Luo, Quan Cheng, Zaoqu Liu, Xinwei Han

**Affiliations:** ^1^ Department of Interventional Radiology The First Affiliated Hospital of Zhengzhou University Zhengzhou Henan China; ^2^ Department of Colorectal Surgery The First Affiliated Hospital of Zhengzhou University Zhengzhou Henan China; ^3^ Center for Reproductive Medicine The First Affiliated Hospital of Zhengzhou University Zhengzhou Henan China; ^4^ Department of Cardiology, West China Hospital Sichuan University Chengdu Sichuan China; ^5^ Department of Urology The First Affiliated Hospital of Zhengzhou University Zhengzhou Henan China; ^6^ Department of Oncology, Zhujiang Hospital Southern Medical University Guangzhou Guangdong China; ^7^ Department of Neurosurgery, Xiangya Hospital Central South University Changsha Hunan China; ^8^ Interventional Treatment and Clinical Research Center of Henan Province Zhengzhou Henan China; ^9^ Interventional Institute of Zhengzhou University Zhengzhou Henan China; ^10^ Institute of Basic Medical Sciences Chinese Academy of Medical Sciences and Peking Union Medical College Beijing China

**Keywords:** CMS classification, colorectal cancer, heterogeneity, molecular subtype, precision oncotherapy

## Abstract

**Background:**

Colorectal cancer (CRC) is among the most hackneyed malignancies. Even patients with identical clinical symptoms and the same TNM stage still exhibit radically different clinical outcomes after receiving equivalent treatment regimens, indicating extensive heterogeneity of CRC. Myriad molecular subtypes of CRC have been exploited for decades, including the most compelling consensus molecular subtype (CMS) classification that has been broadly applied for patient stratification and biomarker‐drug combination formulation. Encountering barriers to clinical translation, however, CMS classification fails to fully reflect inter‐ or intra‐tumor heterogeneity of CRC. As a consequence, addressing heterogeneity and precisely managing CRC patients with unique characteristics remain arduous tasks for clinicians.

**Review:**

In this review, we systematically summarize molecular subtypes of CRC and further elaborate on their clinical applications, limitations, and future orientations.

**Conclusion:**

In recent years, exploration of subtypes through cell lines, animal models, patient‐derived xenografts (PDXs), organoids, and clinical trials contributes to refining biological insights and unraveling subtype‐specific therapies in CRC. Therapeutic interventions including nanotechnology, clustered regulatory interspaced short palindromic repeat/CRISPR‐associated nuclease 9 (CRISPR/Cas9), gut microbiome, and liquid biopsy are powerful tools with the possibility to shift the immunologic landscape and outlook for CRC precise medicine.

## 1 INTRODUCTION

Colorectal cancer (CRC) is one of the most prevalent malignant tumors in the digestive tract.^1^ In 2020, well over 1930,000 cases of CRC were diagnosed globally, and 830,000 deaths were attributed to treatment failure.^2^ Consequently, CRC ranked as the third deadliest cancer and among major causes of cancer‐related deaths worldwide.^3^ The mortality rate pertaining to CRC diagnosis has recently been progressively decreasing with implementations of early tumor screening and refinements in surgical protocols, pre‐operative, and post‐operative care.^4^ Nevertheless, CRC is a model of tumor heterogeneity. Notwithstanding current breakthroughs in nascent therapies, numerous patients still have a dismal prognosis after receiving clinical treatment, resulting in tumor recurrence and even death.^5^ In addition, CRC cases with similar clinical presentations have significant differences in treatment response and survival rate.^6^

For decades, molecular typing analysis of solid tumors, represented by omics exploration has developed rapidly and has been widely carried out in lung cancer,^7^ breast cancer,^8^ gastric cancer,^9^ pancreatic ductal adenocarcinoma,^10^ endometrial cancer,^11^ and so on. CRC‐related molecular typing has also been reported for early diagnosis, treatment, and prognosis assessment of tumors.^12^ For instance, targeted agents such as vascular endothelial growth factor inhibitors and epidermal growth factor receptor (EGFR) inhibitors, in combination with clinical chemotherapy, have been applied to prolong the median survival of patients.^13^ It is also applied in patient stratification, biomarker‐drug co‐development, etc. Since traditional CRC classification could not fully reflect heterogeneity of tumors, the molecular typing of CRC continues to be elusive.^14^ Accordingly, it remains a mission to provide precise treatment for CRC cases with distinguishing features so as to effectively avert under‐ or over‐treatment.

Herein, we systematically summarized molecular typing of CRC, reviewed clinical relevance and application of molecular typing, and further illustrated limitations that could not fully reflect heterogeneity of CRC, which could shed new inspiration on future progress to overcome this straitened circumstance and evolving precision medicine of molecular subtypes in CRC.

## 2 INTRICATE MOLECULAR TYPING IN CRC

### 2.1 Genetic and epigenetic characteristics

#### 2.1.1 Driver events

In 1990, some researchers proposed that occurrence of CRC begins with adenomatous polyposiscoli inactivation mutation followed by activation mutation of oncogene Kirsten rat sarcoma (KRAS) and sequential mutation of SMAD family member four, tumor protein p53 (TP53), and phosphoinositide 3‐kinase catalytic subunit‐*α*. The accumulation of these gene mutations eventually drives CRC carcinogenesis, hence this model is alternatively referred to as “adenoma‐carcinoma sequence model.”^15^ Mutations in several pivotal genes in the model, such as KRAS, TP53, and BRAF, demonstrate prognostic and predictive value and are routinely employed for therapeutic prediction in clinical practice.^16^ For example, patients with the wild‐type KRAS gene are sensitive to targeted therapy with anti‐EGFR monoclonal antibodies (mAbs), whereas individuals with KRAS exon 2 missense mutation are originally resistant to anti‐EGFR mAbs.^17^

However, some CRC cases have significant chromosomal abnormalities and/or epigenetic alterations, but only one of these driving events or none at all.^18^ Only when chromosomal instability (CIN) occurs in conjunction with driver mutations do tumors become aggressive and form large intrahepatic metastases after injection into mice.^19^ In addition, sessile serrated adenomas, which account for 6%–12% of colorectal adenomas, are characterized by high‐frequency BRAF, caudal type homeobox 2 mutations, and epigenetic abnormalities.^20^ This process is termed serrated pathway and is currently considered a far‐reaching supplement to “adenoma‐carcinoma sequence model.”

Incidentally, under impetus of chronic inflammation, normal cells develop into indolent dysplasia, hypoplastic dysplasia, high dysplasia, and eventually cancer. This Inflammatory pathway accounts for <2% of all CRCs. There are palpable and eradicable benign precursor lesions in every pathway, which have a window of opportunity for secondary prevention of CRC since they require several years to evolve into cancer.^21^ In summary, a comprehensive review of genetic and epigenetic characteristics of CRC cells is warranted.

#### 2.1.2 Microsatellite instability

Microsatellite instability (MSI) constitutes approximately 12%–15% of all CRCs.^18^ Genes in DNA mismatch repair (MMR) system malfunction in response to promoter hypermethylation or gene mutations, rendering unrepairable DNA replication errors. Hypermethylation of MLH1 gene promoter is present in bulk of sporadic MSI tumors, and 80%–90% of sporadic hypervariable cancers harbor BRAF^V600E^ mutations.^22^ In contrast, Lynch syndrome tumors predominantly acquire MSI through germline mutation in one of the MMR genes, including MLH1, MSH2, MSH6, and PMS2.^23^ In MSI patients, immunotherapy has become a standard treatment recommended by major guidelines, with excellent responses and outgrowths.^24^, ^25^ Based on the results of KEYNOTE‐177 trial, immune checkpoint inhibitors (ICIs) have been recognized as a first‐line treatment option for metastatic CRC cases with high MSI and/or mismatch repair deficiency (dMMR).^26^, ^27^

#### 2.1.3 CIN

CIN, a hallmark of cancer caused by persistent errors in chromosome separation during mitosis, is common in about 80% of CRCs.^23^ CRC cases with CIN features had poorer overall survival and progression‐free survival compared to patients with MSI features.^28^ However, it fits with “adenoma‐cancer sequential model” best, thus creating an area for CRC chemoprevention. As an example, usage of selective cyclooxygenase‐2 inhibitors was investigated to reduce CRC incidence.^29^

#### 2.1.4 CpG island methylation phenotype

Almost all CRCs have aberrant DNA methylation, whereas 10%–20% of individuals have extraordinarily high CpG methylation frequencies, which refers to CpG island methylation phenotype (CIMP), a CRC epigenetic phenotype.^30^ CIMP is implicated in many downstream events, such as nervous system development, pattern specification, cell signaling, differentiation, and proliferation, which are all involved in cell migration and metastasis.^31^, ^32^ Thus, CIMP may be an underlying target for cancer‐specific therapies.^33^ In accordance with CIMP level, CRC tumors could be categorized into high CIMP group and low CIMP group. Patients with high CIMP phenotypes tend to unveil a poorer prognosis than patients with low CIMP phenotypes.^34^ Even so, there remains controversy about the effect of CIMP on patient prognosis. It has been postulated that CIMP does not correlate with the prognosis of CRC cases excluding the influences of other clinical factors and associated mutations.^35^ With regard to CRC diagnosis, there is an emerging trend to look for aberrantly methylated genes in plasma DNA and fecal DNA as non‐invasive diagnostic tools.^36^

### 2.2 Mutation‐centered classification

Cancer genome sequencing technology, represented by the Cancer Genome Atlas (TCGA), has dramatically improved our understanding of molecular pathways in cancer development and progression.^37^ CRC is one of many tumor types examined in TCGA.^38^ TCGA offered multiple datasets from 276 CRC samples and classified the molecular typing of CRC into three categories. Research has shown that tumor genotype dictates immune phenotype and tumor immuno‐escape mechanisms. Counterintuitively, mutation‐centric classification strategies have nothing to do with, nor do they fully explain the diversity of patient prognosis after specific therapeutic interventions, which may also be affected by tumor microenvironment (TME) and epigenetic alterations.^39^ Accordingly, a more systematic methodology to better stratify CRC cases is needed to overcome limitations of mutation‐based classification schemes.

### 2.3 Classical transcriptional taxonomies and others

Developments in molecular classifications have moved toward more effective interventions and provided vital insights into CRC heterogeneity.^40^ Evidence has emerged over the last decade that transcriptome level best captures intra‐tumor heterogeneity, with a progressive shift in the cancer typing paradigm from a mutation‐centric to a transcriptome‐based approach, which has been successfully applied in gastric cancer, lung cancer, breast cancer, as well as in CRC.^41^ These transcriptome‐based classification systems have unraveled a superior correlation with clinical outcomes, which further makes them more attractive for clinical translation.^39^

Nonetheless, the interactions between genes are ignored in most web‐based approaches, which focus solely on gene nodes in biological networks. Using a program based on individual‐specific gene interaction perturbation networks, six subtypes based on gene interaction networks 1–6 were validated. Gene interaction networks taxonomy raises awareness of CRC heterogeneity based on interaction groups, which lays a foundation for subtype‐based clinical stratification and targeted therapies.^42^

### 2.4 Multi‐omics analysis

Precision medicine is a rising global trend, while identification of novel biomarkers and therapeutic targets is a step forward in such direction.^43^, ^44^ The pathogenesis of CRC involves diverse genetic alterations and numerous pathways. To illustrate, KRAS is one of the most frequently mutated oncogenes in CRC, with approximately 40% of CRC cases exhibiting active KRAS missense mutations.^45^ Carcinogenic KRAS mutations ultimately lead to immune evasion and tumor progression by promoting inflammation and suppressing immune responses.^46^, ^47^ Even so, KRAS mutant tumors remain highly heterogeneous, and further exploration into biological subtypes and molecular targets is warranted to guide prognosis and treatment of patients with KRAS mutant CRC.^48^ This has revealed necessity of multi‐omics analyses for CRC. Comprehensive multi‐omics analyses of genomic, epigenomic, transcriptomic, and proteomic features of CRC could more fully characterize molecular network and biological heterogeneity and refine CRC molecular stratification, which may contribute to development of combination therapies.^45^ In this context, multi‐omics data and integrated analyses are being employed to exploit personalized medicine.^43^ Multi‐omics analyses have had and will continue to make an enormous impact on cancer management, including CRC management.^49^

## 3 PRECLINICAL AND CLINICAL EVIDENCE FOR CRC SUBTYPING

### 3.1 Regulatory factors and potential mechanisms

With regard to CRC, six separate classification systems have recently been reported.^50^ To shed light on promising overlaps and produce more uniform criteria, in 2014, the CRC Subtype Consortium was established to identify four unique consensus molecular subtypes (CMSs) through a web‐based meta‐analysis of six typing systems: CMS 1 (MSI immune type), CMS2 (canonical type), CMS3 (metabolic type), and CMS4 (mesenchymal type) (Table 1).^40^ At present, CMS classification is deemed to be the most persuasive categorization of CRC, on the basis of which many scholars have investigated CRC‐targeted therapies, meaning that it affects prognosis and treatment of CRC in a great sense.^51^, ^52^

**TABLE 1:** Biological characteristics of consensus molecular subtype.

	CMS1 (MSI immune type)	CMS2 (canonical type)	CMS3 (metabolic type)	CMS4 (interstitial type)
Percentage	14%	37%	13%	23%
Genetic characteristics	MSI High mutation rate High‐frequency BRAF mutations Low CIN status	MSS High‐frequency TP53 mutation High SCNAs High CIN status	About 30% have MSI High‐frequency KRAS mutations	MSS High‐frequency TP53 mutation High SCNAs High CIN status
Epigenetic characteristics	CIMP high status	CIMP low status	CIMP low status	CIMP low status
Immune microenvironment	A diffuse immune cell infiltration, like Th1 cells, cytotoxic T cells, and NK cells High expression of immune detection site molecules such as CTLA‐4, PD‐1, PD‐L1, etc. High immunogenicity	Low immune and inflammatory characteristics PD‐L1‐negative Low immunogenicity	Low immune and inflammatory characteristics PD‐L1‐negative Low immunogenicity	Stromal cell infiltration, such as CAFs Inflammatory immune cell infiltration, including Treg cells, MDSCs, monocytes cells, and Th17 cells Pro‐transformation immune escape microenvironment
Transcriptomic pathways	JAK–STAT activation Caspases pathway activation	Epithelial differentiation phenotype Wnt targets MYC activation EGFR and SRC activation Pro‐angiogenesis (VEGF and VEGFR pathways) activation Integrins activation TGF‐β activation	Metabolic reprogramming Including glutaminolysis and lipidogenesis activation	EMT TGF‐β and integrin pathways activation VEGF and VEGFR pathways activation Extracellular matrix remodeling Complement activation
Prognosis	High OS and relapse‐free survival	High OS and relapse‐free survival	High OS and relapse‐free survival	Poor OS and relapse‐free survival

Abbreviations: CAFs, cancer‐associated fibroblasts; CIMP, CpG island methylation phenotype; CIN, chromosomal instability; CTLA‐4, cytotoxic T lymphocyte‐associated antigen‐4; EGFR, epidermal growth factor receptor; EMT, epithelial‐mesenchymal transition; JAK–STAT, Janus kinase‐signal transducer and activator of transcription; KRAS, Kirsten rat sarcoma; MDSCs, myeloid‐derived suppressor cells; MSI, microsatellite instability; MSS, microsatellite stable; MYC, myelocytomatosis; OS, overall survival; PD‐1, programmed cell death protein 1; PD‐L1, programmed cell death ligand 1; SCNAs, somatic copy number aberrations; TGF‐β, transforming growth factor‐β; Th 1, T helper cell 1; TP53, tumor protein p53; Treg, regulatory T cells; VEGF, vascular endothelial growth factor; VEGFR, vascular endothelial growth factor receptor.

In total, 235 functional gene regulators (FGRs) were identified by integrating genome, epigenome, transcriptome, and interactome of CMSs, including chromatin regulators, RNA‐binding proteins, and transcription factors. Certain cancer‐related pathways are highly correlated with FGRs, including those involved in cell growth and death, signal transduction, cell cycle, and DNA replication, particularly all genetic information processing pathways in four CMS subtypes. Moreover, anti‐tumor immune pathway of CMS1 is mainly activated by FGRs.^53^ To illustrate, it has been investigated that expression of signal transducer and activator of transcription 1 (STAT1) in CMS1 pertaining to high infiltration level of CD8^+^ T cells and activation of T cell receptor signaling pathway, which drives a hyper‐immunogenic TME and strong anti‐tumor immune cell infiltration in CMS1, while the others displayed adverse immunogenicity.^54^ Other FGRs, comprising STAT4, IRF4, and SOX11, have also been demonstrated as a nexus with immune regulation in CMS1. Overall, FGRs synergistically modulate cancer‐related pathways and TME in CMSs, suggesting latent targeting strategies to enhance treatment outcomes for relevant subtypes, which has led us to optimize CMS classifier.^53^

### 3.2 Exploring subtypes through cell lines and animal models

Different CRC molecular subtypes arise from different molecular disease mechanisms, related to distinct clinical outcomes.^55^ Cell lines are fundamental to discovering nascent anti‐tumor agents and biomarkers. Clinical efficacy has been demonstrated among patients with molecular markers in response to conventional chemotherapy and targeted drugs.^56^, ^57^ Moreover, the presence of four CMS subtypes has been demonstrated in in vitro model systems, thus cancer cell‐intrinsic aberrations characteristic of four CMS groups could be identified using cancer cell lines.^58^ CRC cell lines have been elucidated to recapitulate molecular alterations and pharmacogenomics of primary tumors as measured by genomic studies and drug sensitivity screening.^59^, ^60^ Hence, for further studies of intrinsic differences between cancer cells, functional biological mechanisms, and pharmacogenomics of CRC, CRC cell lines provide an indispensable resource, which augments their value as preclinical models of CRC.^58^

Animal models have also been applied in CRC subtyping. CRC subtypes are defined by colon location, differing genetic mutations, genomic instability, pathology, and epigenetic biomarker profiles, which are potentially orchestrated by microbiota, and are recognized as a key contributor to disease.^61^, ^62^ Enterotoxigenic *Bacteroides fragilis* (ETBF) colonization is connected with CRC cases and tumors.^63^ In an attempt to examine role of microbes in CRC, an ETBF‐colonized multiple intestinal neoplasia (Min) mouse model was previously established, which was investigated and characterized by interleukins (IL)‐17‐dependent distal colon adenomas. In comparison, after addition of BRAF^V600E^ mutation to Min mouse model, called ETBF‐colonized BRAF^V600E^ Lgr5^Cre^ Min mouse model, results were typified by a mid‐proximal colon tumors emergence, CpG island DNA hypermethylation, high infiltration of CD8^+^ T cells, interferon (IFN)‐γ signatures expression, and sensitive to anti‐programmed cell death ligand 1 (PD‐L1) treatment, which is consistent with findings in melanomas from patients that responded to anti‐programmed cell death protein 1 (PD‐1) checkpoint therapy.^64^ These results provide latest insights into interactions between genes and microbiota, which may be relevant to pathogenesis of BRAF^V600E^‐mutated CRC in humans and are crucial to guiding targeted therapies in CRC.^65^

### 3.3 Patient‐derived xenografts and organoids models

Patient‐derived xenograft (PDX) model maintains biological characteristics of native tumors, which perform an instrumental role in personalized medical decision‐making.^66^ PDXs have also been executed in the field of CRC to replace human tumor stromal cells with their mouse counterparts. Thereby human‐specific expression profile of CRC PDXs was applied to assess intrinsic transcriptional profile of cancer cells so as to exclude influence of tumor stromal components, which helped identify five CRC intrinsic subtypes endowed with distinctive molecular, functional, and phenotypic peculiarities, refining biological insight into CRC heterogeneity.^67^

Organoids are three‐dimensional structures, miniaturized in vitro organ models that highly mimic genetic and epigenetic characteristics of target tissues or organs in vivo.^68^ Accordingly, organoids have been widely applied in organ development, precision medicine, regenerative medicine, and so on, serving as a valuable tool for researching a wide range of biological processes and diseases.^69^, ^70^, ^71^ Furthermore, organoids are also being employed for CRC typing. For instance, epigenetic pattern alterations are a driving force behind tumor heterogeneity and carcinogenesis.^72^ Loss of trimethylation of H4K20 is linked to shorter survival and elevated tumor recurrence rates in CRC.^73^ To comprehend epigenetic regulation in CRC, patient‐derived organoids and mouse intestinal organoids were genetically engineered.^74^ Results manifest that H4K20 trimethylation deletion mediated by lysine methyltransferase SUV420H2 is discovered to facilitate right‐sided colorectal tumorigenesis over chromatin compaction epigenetically controlled, which unravels a promising avenue for CRC subtype‐specific therapy.^74^ In the context of precision therapy, advantages of organoids are rapidly making them an essential tool for individualized treatment selection.^75^

### 3.4 Clinical trails

Over the past decade, stratifying patients into distinct molecular subtypes has been achieved in CRC.^76^ By way of example, in 2013, primary tumor samples in seven centers from a large multicenter cohort of 566 patients who underwent surgery between 1987 and 2007 were included to establish a comprehensive molecular classification of colon cancer (CC). Using a discovery subset of 443 CC samples, six molecular subtypes with varying molecular characteristics were identified by Marisa et al. (Table 2).^77^ The classification provides a basis for designing robust prognostic features, as well as determining specifically targeted markers for distinct CC subtypes.

**TABLE 2:** Main characteristics of colon cancer molecular subtype system.

	C1 (CINImmuneDown)	C2 (dMMR)	C3 (KRASm)	C4 (CSC)	C5 (CINWntUp)	C6 (CINnormL)
Frequency	21%	19%	13%	10%	27%	10%
Genome Instability	High frequency of CIN	High frequency of dMMR High‐frequency CIMP	Intermediate frequency of CIN	Intermediate frequency of CIN and CIMP	High frequency of CIN	High frequency of CIN
Mutations	High enrichment for KRAS TP53 mutations	High enrichment for KRAS and BRAF mutations	Significant enrichment for KRAS mutations	High enrichment for KRAS mutations	High enrichment for KRAS TP53 mutations	High enrichment for KRAS TP53 mutations
Signatures		Significant enrichment for upregulated genes in BRAFm‐like and Serrated CC‐like signatures Intermediate enrichment for upregulated genes in normal‐like signatures	Intermediate enrichment for upregulated genes in serrated CC‐like signatures	Significant enrichment for upregulated genes in SC‐like and serrated CC‐like signatures; Intermediate enrichment for upregulated genes in normal‐like and BRAFm‐like signatures		Significant enrichment for upregulated genes in normal‐like signature; Intermediate enrichment for upregulated genes in serrated CC ‐like signatures
Pathways	Downregulated immune system and EMT	Upregulated immune system Upregulated proliferation pathway Downregulated Wnt pathway	Downregulated immune system and EMT	Upregulated EMT Downregulated proliferation pathway	Upregulated Wnt pathway	Upregulated EMT Downregulated proliferation pathway

Abbreviations: CC, colon cancer; CIMP, CpG island methylation phenotype; CIN, chromosomal instability; dMMR, mismatch repair deficiency; EMT, epithelial‐mesenchymal transition; KRAS, Kirsten rat sarcoma; SC, satellite cell‐like; TP53, tumor protein p53.

In bulk masses of solid tumors, tertiary lymphoid structure (TLS) is regarded as a predictor of favorable prognosis.^78^ Intriguingly, prognostic value and relevant mechanisms of TLSs in colorectal cancer liver metastases (CRCLM) were evaluated by a clinical trial, and 603 patients with CRCLM treated by surgical resection from three cancer centers were enrolled.^79^ It demonstrated that CRCLM survival and transcriptomic subtypes were closely correlated with distribution and abundance of TLSs. Thereby, to predict the prognosis of CRCLM patients, the latest immune class has been proposed. TLSs could contribute to clinical immunotherapy response levels in CRCLM. As mentioned above, pembrolizumab evinced a better objective response rate and progression‐free survival compared to chemotherapy in KEYNOTE‐177 study, which randomized patients with high MSI and/or dMMR metastatic CRC to first‐line treatment.^80^ Overall, clinical trials have been extensively conducted in field of CRC, which significantly fosters evolution of CRC typing and precision treatment.

### 3.5 Targeted drug development strategies

#### 3.5.1 From “One gene, one drug” to “Multi‐gene, multi‐molecule, multi‐drug” model

**FIGURE 1:**
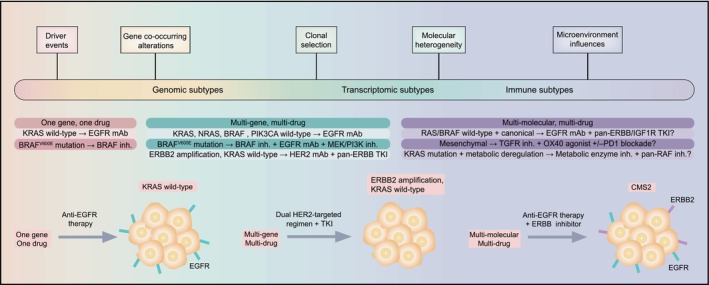
Evolution of precision medicine paradigms in CRC. The increased understanding of CRC biology has facilitated a shift in the treatment paradigm from “One gene, one drug” and “Multi‐gene, multi‐drug” to “Multi‐gene, multi‐molecular, multi‐drug,” which contributes to advances in biomarker‐drug combination development. HER2, human epidermal growth factor receptor 2; IGF1R, insulin‐like growth factor 1 receptor; inh., inhibitor; MEK, mitogen‐activated protein kinase; PIK3CA, PI3K catalytic subunit‐α; TKI, tyrosine kinase inhibitor.

A stratified model for KRAS gene mutation has ushered in the era of CRC precision therapy, also known as “One gene, one drug” model.^51^ Studies have revealed that therapeutic benefits of EGFR mAbs are limited to patients with wild‐type CRC at all KRAS and NRAS loci.^81^ However, the majority of KRAS wild‐type patients were equally insensitive to EGFR inhibitors cetuximab and ranibizumab, suggesting additional resistance mechanisms, involving mutations in BRAF, MEK1, ERBB2, FGFR1, and PDGFRA, as well as a crowd of drawbacks to single‐site targeted therapy.^82^, ^83^, ^84^, ^85^ Based on advances in our understanding of CRC genomic and transcriptomic subtypes and beyond, we present a classification system that combines molecular characteristics and targeted drugs, transitioning from a “One gene, one drug” to a “Multi‐gene, multi‐molecule, multi‐drug” model in treatment decision‐making, which has impacts on biomarker‐drug co‐development (Figure 1).^51^

For example, in patients with ERBB2 amplifications, dual HER2‐targeting regimens in combination with tyrosine kinase inhibitors have shown significant clinical activity.^86^ In addition, anti‐EGFR targeted therapy typically prolongs survival time in CMS2 tumors due to high‐frequency amplification and/or overexpression of EGFR ligand and insulin receptor substrate 2.^87^, ^88^ Nevertheless, ERBB2 and insulin‐like growth factor 2 genes are also amplified in CMS2 tumors, which potentially drive resistance to EGFR mAbs.^89^ Correspondingly, a combination of ERBB and insulin‐like growth factor 1 receptor inhibitors may get more effective results.^51^ Besides, in patients whose tumors displayed high integrin‐αvβ6 expression levels, combination therapy with cetuximab and a mAb anti‐integrin‐αv was particularly effective.^90^

#### 3.5.2 Immune‐sites targeted therapy

Cancer progression relies on dynamic interactions between a spectrum of cell types, notably cancer cells and immune cells.^91^ Clinical outcomes of CRC are modulated by immune response.^92^ Chemotherapy, radiation, and surgery are typical therapeutic modalities exerted in embryonic stages of CRC, which lead to a sea of problems such as toxicity and drug resistance.^93^, ^94^ In this context, researchers are constantly looking for better therapeutic strategies for CRC cases. In recent years, ICIs have emerged as a new therapeutic approach with remarkable results in hematological malignancies and solid tumors.^95^ Blocking PD‐1/PD‐L1 pathway with ICIs has been successful in altering interactions between immune system and cancer toward rejecting or inhibiting tumor progression.^96^ However, immune checkpoints enable the immune system to maintain an equilibrium between protection against pathogens and disturbances in autoimmunity. While ICIs result in T‐cell activation, excessive activation of T cells may give rise to immune‐related adverse events such as auto‐immunity.^97^ These adverse events are well controlled clinically, but in very few cases they could be serious and lethal. Advanced studies on molecular mechanisms of adverse reactions are required to manage better and prevent side effects.^98^

**FIGURE 2:**
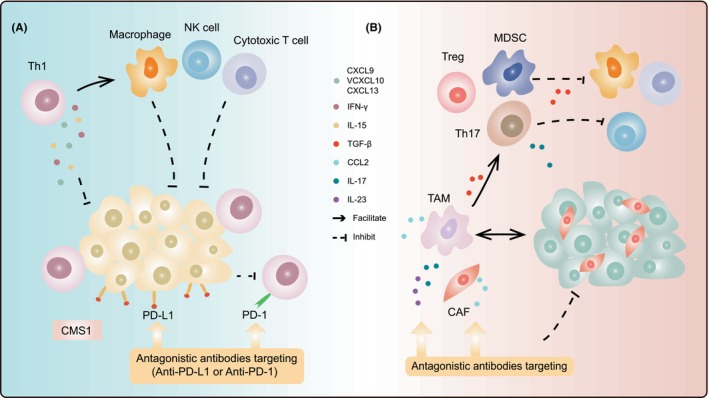
Immune characterization of colorectal cancer and essential targets for immunotherapies. (A) CMS1 (MSI immune subtype) is characterized by diffuse immune cell infiltration (e.g., Th1, cytotoxic T cells, and NK cells); high expression of immune detection site molecules (e.g., CTLA‐4, PD‐1, PD‐L1) in tumor cells and tumor‐infiltrating immune cells; high expression of IFN‐γ, IL‐15, CXCL9, CXCL10, and CXCL13. The cumulative interaction of PD‐1 with PD‐L1 results in a state of T cell dysfunction often referred to as T cell exhaustion. (B) CMS4 is characterized by stromal cell infiltration (e.g., CAFs); high immunosuppressive cell infiltration (e.g., Treg, MDSCs, and Th17); significant upregulation of immunosuppressors (TGF‐β, CXCL12, CCL2, IL‐23, and IL‐1). CCL, chemoattractant cytokine ligand.

Admittedly, TME also has a hand in recruitment and activation of cytotoxic immune cells, which comprises a constellation of immunomodulatory cytokines/chemokines and suppressive immune cells, thus, determining responsiveness of CRC to immunotherapeutic agents.^99^ As proof, CMS1 is characterized by high infiltration of cytotoxic T cells and Th1 cells, and upregulation of multiple immune checkpoints.^100^ Cytotoxic T lymphocyte‐associated antigen‐4 (CTLA‐4), PD‐1, PD‐L1, and indoleamine 2, 3‐dioxygenase 1 are highly upregulated in CMS1. These tumors also have the highest expression of genes involved in Th1 phenotypic orientation (e.g., IFN‐γ and IL‐15), TLS formation (e.g., C‐X‐C motif chemokine ligand 13 (CXCL13)), chemokines attracting T cells (e.g., CXCL9, CXCL10, and CXCL16), and activate Janus kinase‐STAT pathway (Figure 2A).^40^ CMS4 is labeled by high expression of regulatory T cells (Treg), myeloid‐derived suppressor cells (MDSCs), and Th17. Significant upregulation of immunosuppressors (transforming growth factor‐β (TGF‐β), CXCL12, CCL2, IL‐23, and IL‐17) suppressed cytotoxic immune cells via chemokines and favored proliferation of MDSCs, B cells, and Tregs (Figure 2B).^51^, ^101^, ^102^ For upregulation or downregulation of miscellaneous molecules mentioned above, corresponding targeted drugs could be found to restrain neoplastic progression and achieve individualized precision therapy.

## 4 CURRENT CHALLENGES AND FUTURE DIRECTIONS FOR CRC PRECISION THERAPY

### 4.1 Current challenges of CRC precision therapy

#### 4.1.1 Challenges impeding CMS clinical practice

The CMS classification system offers a reference for current researchers to understand carcinogenic effects, cancer progression, and drug resistance of CRC. Encouragingly, it provided the most robust classification system for CRC, with a clear molecular basis and clinical relevance, which not only lays foundation for further research on subtype‐specific biological mechanisms but also impetus for clinical translation into more optimal treatment and latest targeted drug formulation.^39^ Even so, it has some limitations simultaneously.

First and foremost, in current clinical practice, mutation status is pivotal to selecting chemotherapy drugs, while CMS is developed based on gene expression.^103^ On top of that, although a range of CRC‐targeted therapies are based on CMS classification, with a deeper comprehension of CRC typing, there has been a recognition that disparate CMS subtypes have significant biological differences, accordingly leading to distinct drug reactivity.^104^ Based on comprehensive analyses of mutations and copy number variations from TCGA data, BRAF mutations are regularly observed in CMS1 and are associated with MSI phenotype. KRAS mutations are routinely encountered in CMS3. In addition, receptor tyrosine kinase pathways and mitogen‐activated protein kinase pathways are commonly activated in CMS1 and CMS3. Nonetheless, these aberrations are not specific to any of CMS subtypes. The heterogeneity of CMS status observed in CRCs with recognized driver gene events highlights notable variability in biological behavior of such tumors.^40^ Moreover, TME strongly impresses CMS classification, as evidenced by high expression of mesenchymal marker genes in stroma of tumors of stem‐like/mesenchymal subtype CMS4.^105^ In conclusion, CMS classification has different immunological, stromal, and clinicopathological features, which exhibit significant intra‐tumor heterogeneity. Further efforts are needed to fully assess functional roles of CMS.

#### 4.1.2 Stratified management of clinical patients

Concerning CC, consensus immunoscore has proven to be a reliable prognostic indicator independent of the TNM staging system.^106^ Immunoscore, a tissue‐based assessment of memory and cytotoxic tumor‐infiltrating lymphocytes at the margins or center of cancer infiltration based on CD3 and CD8 markers, was identified by Galon et al.^107^ Immunoscore has been established as the most powerful prognostic predictor of CRC, even superior to TNM staging.^108^ Whereas a number of other cellular elements of TME may also play a role in tumor occurrence and progression. Clinical importance of TME characteristics in an army of cancer types has been recognized and highlighted.^109^, ^110^ Numerous studies have illuminated that matrix and immune markers such as fibroblasts and cytotoxic T cells may be critical drivers underlying clinical outcomes.^106^ Cancer‐associated fibroblasts (CAFs), immunosuppressive TME, and extracellular matrix sclerosis are identified as major contributors to chemotherapy resistance in treatment of CRC.^111^ To some extent, these factors have obstructed advancement of immunotherapy. A deeper insight into dormant mechanisms of CRC drug resistance remains an unmet need. Given this, single‐cell characterization was carried out to explore molecular composition of tumor and TME in each CMS. It was observed that CMS4 subtype attested to obvious infiltration of CAFs and C1Q^+^ tumor‐associated macrophages, which are strongly connected with low disease‐free survival, cancer growth, and immunosuppressive microenvironments. This result indicates that stratifying patients depending on CAF subtype characteristics and targeting these subtypes may be next step toward stratified clinical patient management, novel combination therapies, and subtype‐specific therapies.^14^ Still, we believe that other cell types may also play a part in CRC biology. Future studies should aim to better understand CAFs and other cell subtypes prevalent in CRC.

#### 4.1.3 Heterogeneity barriers of CRC

**FIGURE 3:**
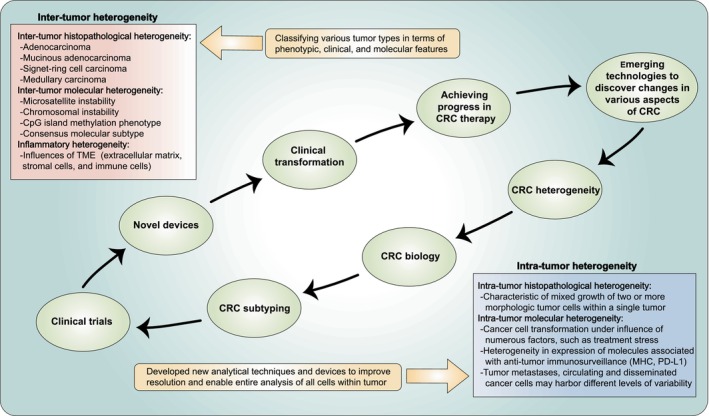
Heterogeneity in CRC and the future directions. CRC is a complex disease with strong heterogeneity both between and within tumors. Emerging technologies will be needed in the future to discover alterations in CRC genome, epigenome, transcriptome, secretome, metabolism, etc., which will help to evolve a deeper understanding of CRC heterogeneity and biology. Based on this, it will be possible to develop more precise CRC subtypes. Furthermore, these subtypes should all be tested in appropriately designed clinical trials, and further they could be applied to facilitate clinical decision‐making through elaboration of new devices if these subtypes dissect exploitable value in clinical trials, which would be a significant step toward implementation of precision medicine for CRC. MHC, major histocompatibility complex.

Throughout the above, it is straightforward to conclude that CRC is highly heterogeneous. The development of high‐throughput sequencing techniques allows detection of genomic, epigenomic, and transcriptomic variations related to development and evolution of CRC, which accentuates inter‐tumor heterogeneity.^112^ Intra‐tumor heterogeneity exists in almost every cancer type, which affects tumor progression and clinical prognosis.^113^ Nevertheless, numerous molecular subtypes of CRC could hardly address intra‐tumor heterogeneity for ensuing reasons. To begin with, subtypes are not absolute for individuals, which solely overexpress the most dominant characteristics. To be more specific, subtypes could be transformed under curative and alternative pressures. Following chemoradiotherapy, for instance, CMS4 may morph into CMS1. In light of preceding reasons, due to distinctive intrinsic characteristics of patients, the same treatment regimen for them with the same subtype could engender completely disparate treatment results, contributing to ineffective or drug‐resistant treatments. Although genetic tumor heterogeneity could function in tumor progression, how intra‐tumor heterogeneity informs CRC stratification and outgrowths remains unclear.^114^ Nowadays, recent advances in single‐cell analysis and spatial technology have further revealed complexity of tumor ecosystems.^115^ These emerging technologies have potential to illuminate inter‐ and intra‐tumor diversity in CRC, with ramifications for selection of specific molecular biomarkers and clinical decision‐making (Figure 3). Novel biomarkers that are not present in healthy individuals but are available in CRCs are still being explored, particularly those that could be measured in early stages of disease initiation and used in diagnostic tests. Regrettably, no molecules have been identified to date that fulfill all of the above criteria. Carcinoembryonic antigen is as yet the only acknowledged tumor marker for monitoring patients in the course of CRC treatment, both during and after treatment.^116^

### 4.2 Future directions of CRC precision therapy

#### 4.2.1 Nanotechnology in CRC diagnosis and treatment

There are bulk masses of unique and amazing nanomaterials that have promising diagnostic and therapeutic applications in CRC. Miscellaneous nanomaterials have been verified in cancer biology and could be utilized as a revolutionary avenue to facilitate CRC diagnosis and treatment because of their strong specificity and long blood circulation time.^117^ The primary advantage of nanoparticles (NPs) is their compact size, which benefits efficient diagnostic drugs and targeted drug delivery.^118^ Inorganic NPs, such as carbon nanotubes harbor utility as enhancement of imaging techniques owing to their excellent stability and minimal biodegradation for diagnostic use. Organic NPs including but not limited to liposomes are less robust but more biocompatible for drug delivery. Conventionally exerting therapeutic drugs in cancer treatments could compromise the immune system and trigger a multitude of side effects. Comparatively, nano‐drug delivery systems minimize previous side effects by encapsulating therapeutic chemicals and delivering tailored drugs into tumor niches, which not only reduces toxicity in vivo, but also offers high stability, biocompatibility, and effectiveness.^119^

Encouragingly, curcumin has been nanosized into micelles, nanogels, liposomes, NPs, and cyclodextrins for CRC treatment.^120^ NPs could augment effectiveness of chemotherapy by targeting tumor cells in particular, which have higher bioavailability, better tissue targeting, and fewer side effects than free drugs.^121^ Targeted NPs bind to a spectrum of active substances, such as chemotherapeutic drugs, and RNA molecules to silence genes, proteins, and contrast agents.^122^ Targeted modified NPs assist with increasing concentration of drugs in tumor tissue, facilitating specific delivery to tumor and preventing release of drugs into general population, improving pharmacokinetics and pharmacodynamics of drugs, and overcoming tumor cell resistance mechanisms.^123^ New therapies using nanomaterials for treatment of CRC may become more clinically available. However, despite the great potential of these nanotherapeutic systems, there are some issues regarding biodistribution, localization improvement, biocompatibility, and effectiveness in real‐time treatment of CRC in vivo.^119^ Although the field has not yet produced a unified view of nanotechnology for CRC diagnosis and treatment, there is a partial consensus that introduction of NPs into drug delivery has a profound impact on favoring standard of living and survival for CRC patients.^119^

#### 4.2.2 Clustered regulatory interspaced short palindromic repeat/CRISPR‐associate nuclease 9 (CRISPR/Cas9) for CRC

The CRISPR/Cas9 system is a formidable gene editing technology that boosts studies of oncogenes, tumor suppressor genes, drug‐resistant genes, target genes, mouse model construction, and especially genome‐wide library screening. CRISPR/Cas9 technology is adept at speed, simplicity, and fidelity in head‐to‐head comparisons with other traditional gene editing tools.^124^ In 2020, CRISPR/Cas9 technology was extensively exploited to construct human serrated adenoma models elucidating RSPO fusion genes and GREM1 overexpression in CRC.^125^ Proverbially, CRISPR/Cas9 gene editing technology is demonstrated as a nexus with multiple organoid species to unveil tumor and drug resistance genes, which optimizes/drawbacks of traditional cell line culture, as well as better mimics biological behavior of tumors in sophisticated milieus.^126^, ^127^, ^128^ Encouragingly, a few researchers employed CRISPR/Cas9 knockout libraries to screen proto‐oncogenes, tumor suppressor genes, and tumor resistance genes for constituting xenograft mouse tumor models.^129^

Besides, the CRISPR/Cas9 system could unravel mechanisms of inherited CRCs and bolster accurate diagnosis, genetic counseling, and prevention of CRC hereditary. Moreover, the CRISPR/Cas9 system has been evolved into gene therapy of CRC, which implicated in molecularly targeted drug delivery or in vivo targeted knockout. Systemic administration of the CRISPR/Cas9 system has been manifested to strikingly throttle CRC growth in xenograft mice and handicap CRC‐induced liver and lung metastases.^130^ The benefits of CRISPR/Cas9, as mentioned above, have made contributions to different aspects of CRC research. And yet, its application is still restricted owing to its drawbacks such as off‐targeting and limited delivery methods. It is evident that more studies are warranted on gene therapy for CRC patients.^131^

#### 4.2.3 Organoids for CRC modeling, decoding, and targeting

Patient‐derived organoids are akin to biology of tissues and tumors, validating ex vivo human disease modeling and dissecting main characteristics of intra‐ and inter‐tumor heterogeneity. Organoids could be extensively utilized to compare tumors with normal tissue or to assess cytotoxic effects on healthy tissues.^132^ Promisingly, integrity of tumor genome signature is sustained in organoid cultures.^133^ Furthermore, organoids preserve intra‐tumor heterogeneity and stem cell hierarchies with differentiation trajectories in human tumors, shedding light on tumor heterogeneity and plasticity.^134^

In recent years, organoids have been engineered as models for drug discovery and guiding clinical decision‐making. Until very recently, a versatile combination of single‐cell sequencing and tumor slice culture offered renewed directions for validating chemotherapy response and elucidating two distinct subtypes of CRC liver metastases, which exhibit different immune checkpoint ligands and respond disparately to chemotherapy.^135^ In terms of CRC, these advances allow generation of biospecimen libraries and personalized models with functional insights into treatment response and resistance mechanisms.^75^, ^136^ We focus on the role of organoids in decoding cancer cell dynamics and complexity of TME, along with potential bi‐directional crosstalk.

A multitude of studies manifest powerful opportunities that organoids provide for exerting disease models to better validate clonal evolution and identify functional CRC drivers which is of great concern for CRC patients.^129^, ^137^, ^138^ Expectedly, organoids will be applied in decoding epigenetic regulation of tumor cell plasticity in response to progression and drug resistance. Moreover, organoids will put forward new inspiration on the role of stagnant cells and dormant state cells in obstructing cancer recurrence in patients. Organoid technology has massively scaled up a number of cancer models as well as genetic and phenotypic diversity. In addition, advances in living biobanks, screening methods, organoid‐based precision medicine, and challenges of co‐clinical trials are reinforced.^139^ Wetterling et al. were first to engineer a prospective “living biobank” of CRC organoids.^140^ Concomitantly, further biobank and drug screening platforms have been defined for organoids derived from CRC and its metastases, in conjunction with other tumor entities.^141^, ^142^, ^143^, ^144^

Rae and colleagues have recently reviewed their limitations, incorporating more expensive than two‐dimensional culture, time‐consuming, high‐throughput screening not fully developed, and limited availability of expertise. We need to go one step further in search for ideal tumor models.^145^

#### 4.2.4 Gut microbiome in CRC clinical diagnosis and treatment

CRC transforms from polyps and adenomas to malignant tumors, which are affected by a vast majority of genetic and environmental factors including but not limited to dysbiosis. Much of metagenomic studies of CRC have examined dysbiosis of intestinal flora as a principal risk factor in evolution of colorectal malignancies. A growing body of evidence demonstrates that gut microbiome endows CRC susceptibility, interacts directly with tumors, or modulates patient response to chemotherapeutic and immunotherapeutic agents. Bacteria in colon frequently interact with colonic epithelial cells and other microorganisms to regulate physiological processes comprising energy exchange and host immunity.^146^, ^147^ Dysbiosis of intestinal flora, such as enrichment of various bacterial groups like asusobacterium nucleatum, Peptostreptococus anaerobic, and ETBF, has been implicated as a cause of CRC carcinogenesis.^148^

The close connection between normal physiology and gut microbiome has been gradually recognized, which substantiates pathologic imbalance in gut microbiome and is pertaining to tumorigenesis and progression.^149^ Mechanisms by which gut microbiome is involved in development of CRC consist of genotoxic effects of pathogenic bacteria, immune modulation by the gut microbiome, and microbial metabolome and CRC. Encouragingly, analysis of microbial communities in fecal and mucosal samples has elucidated that specific changes in gut microbiome are implicated in different stages of CRC, unveiling diagnostic potential of gut microbes in CRC detection.^150^ Given the paramount role of gut microbes in CRC, targeting gut microbiome is a powerful tool with possibility to shift immunologic landscape and outlook for the sake of acquiring optimal therapeutic approaches.^151^ To provide inimitable insights into therapeutic interventions, diet, and lifestyle that could alter gut microbiota and associated metabolites to augment CRC. Gut microbiome modulates responses to cancer chemotherapy through multiple mechanisms ranging from immunomodulation, translocation, and enzymatic degradation. Chemotherapeutic agents orchestrate TME and spur tumor‐damaging immune responses via commensal bacteria which could induct advances in composition and gene expression of gut microbiome.^152^

Yet, these findings are only the tip of iceberg regarding uncharted information about gut microbiota. A more thorough comprehension of CRC microbiota necessitates establishment of easy‐to‐use tools that will allow us to perform metagenome‐wide strain‐level analyses to discern genetic variability in gut microbes and to explore formerly unannotated portions of gut microbiota.^153^ In a nutshell, interactions between gut bacteria, cancer immunity, and therapy remain relatively vague and controversial, utmost efforts should be attached to unveiling gut microbiomes to reveal complex compositional changes linked to CRC.

#### 4.2.5 Liquid biopsy in CRC precision medicine

Frustratingly, a multitude of CRC cases respond poorly to conventional treatments, and CRC survival is markedly modulated by primordial diagnosis and early treatment, there is an urgent need for a known biomarker to early predict beneficial responses. Tissue biopsy is one of the most powerful tools with possibility for tumor identification, whose major shortcoming is repeated injuries and weak patient compliance incurred by frequent biopsies. Accordingly, it has been reconciled with current paradigm into a minimally invasive or non‐invasive means to filter high‐risk populations and monitor presence of CRC in asymptomatic patients at an embryonic and curable stage. Admittedly, liquid biopsy could identify circulating cancer‐derived biomarkers to differentiate cancer cells released from primary tumor and/or metastatic sites.^154^, ^155^ Accumulating data substantiates that liquid biopsy is non‐invasive, mitigates tumor heterogeneity, and harbors real‐time critical care monitoring in tumor progression, recurrence, or response to treatment.^156^

Circulating tumor DNA (ctDNA), circulating tumor cells (CTCs), circulating tumor RNA, and exosomes are profound target components studied in liquid biopsies.^157^ Unlike other cancer biomarkers, CTCs are cancer cells that could deliver biological and molecular evidence of cancer cells supporting single‐cell analysis and directly indicate ongoing changes in cancer cells at all different phases of disease.^158^, ^159^ Within CRC patients, CTC pool may comprise more than just epithelial tumor cells, but also tumor cells from epithelial‐mesenchymal transition and cancer stem cells (CSCs).^160^ CSCs are a subset of tumor cells that drive tumorigenesis and lead to recurrence.^161^ While most CRC patients have tumors that could be surgically removed, a large percentage of these patients will ultimately die from metastatic disease.^162^ To illustrate, minimal residual disease (MRD) is defined as continuation of cancer in a patient after treatment, indicating any tumor cells that have spread from primary lesion to distant organs, or any tumor cells that remain after therapy and ultimately result in local recurrence, which is undetectable by modern medical imaging methods, and represents an insidious stage in advancement of cancer. Liquid biopsy methods based on measurement of trace amounts of CTCs or ctDNA have made astonishing breakthroughs in detecting MRD in patients with a variety of malignancies.^163^

A single‐center study by Dalum et al. noted that CTC measured 2–3 years postoperatively portrayed an unfavorable prognosis, suggesting that long‐term persistence of MRD is an indispensable prerequisite for CRC recurrence.^164^ Further characterization of CTC and ctDNA can provide insights into the molecular evolution of MRD during tumor progression, which is of great significance in delaying or even preventing the treatment of metastatic recurrence.^165^ Given their importance, a sea of biomarkers characterizing CSCs (e.g., CD44, CD166, EpCAM, Oct‐3/4) have been established and linked to CRC diagnosis, treatment, and prognosis.^166^, ^167^, ^168^ Yet, studies have shown that CSCs are highly plastic and could change their phenotype and functional outlook. There is growing evidence that CSCs are dynamic populations, which could shift between quiescence and multiplication due to plasticity.^169^ It is thus not astonishing that existing anti‐CSC strategies targeting preclinical stemness‐associated factors have been discouraging. Therefore, there is a huge opportunity to exploit new strategies targeting circulating CSCs for CRC treatment.^170^

Another paramount element of liquid biopsy is exosomes, which act as a latent agent in tumor initiation, progression, and metastasis and are also easier to isolate than CTCs in tumors. A great deal of work has been done to detect and treat CRC using isolation and characterization methods of CTCs, exosomes, and ctDNA, which have been manifested to be highly sensitive and effective. Herein, we summarize various trials using CTCs including the COBRA trial in CRC (Table 3).

**TABLE 3:** Summary of clinical trials of CTCs in CRC.

NCT number	Phases	Status	Tumor types	Interventions	Study results
NCT05350501	II	Completed	CRC	Drug: EO2040	–
NCT04917276	–	Not yet recruiting	CRC Stage IV CTC	Diagnostic test: CTC	–
NCT04912882	–	Not yet recruiting	CRC CTC	Diagnostic test: CTC	–
NCT04842006	–	Recruiting	CRC	Drug: total neoadjuvant therapy Diagnostic test: MRD Radiation: long radiation therapy	–
NCT04775862	II	Unknown	CC	Drug: investigator choice re‐challenge with anti‐EGFR Rx	–
NCT04258137	–	Recruiting	CRC NSCLC	Genetic: liquid biopsy	–
NCT04068103	II | III	Active not recruiting	Colon adenocarcinoma Stage IIA CC AJCC v8	Drug: capecitabine Drug: fluorouracil Drug: leucovorin Drug: leucovorin calcium Drug: oxaliplatin Other: patient observation	–
NCT03844620	II	Active not recruiting	Refractory colorectal carcinoma Stage III CRC AJCC v8 Stage IIIA CRC AJCC v8 Stage IIIB CRC AJCC v8 Stage IIIC CRC AJCC v8 Stage IV CRC AJCC v8 Stage IVA CRC AJCC v8 Stage IVB CRC AJCC v8 Stage IVC CRC AJCC v8	Other: best practice Other: laboratory procedure Other: quality‐of‐life assessment Other: questionnaire administration Drug: regorafenib Drug: trifluridine and tipiracil hydrochloride	–
NCT03551951	–	Recruiting	NSCLC Esophageal cancer Gastric cancer Pancreatic cancer Hepatocellular cancer CRC	Diagnostic test: test for CTCs DNA alterations	–
NCT03476122	–	Unknown	Colorectal cancer	–	–
NCT03295591	–	Unknown	Metastatic CRC CTC	Diagnostic test: CTC	–
NCT03256084	–	Recruiting	CRC	Procedure: blood and tumor samples	–
NCT02979470	–	Unknown	CC Stage IIb Rectum cancer Adenocarcinoma CC Stage IIIa CC Stage IIIc CC Stage IIIb	–	–
NCT02955173	–	Completed	CTCs CRC Gastric cancer	Procedure: surgery	–
NCT02948985	–	Unknown	CRC metastatic	Other: treated with FOLFIRI±cetuximab	–
NCT02874885	–	Active not recruiting	Locally advanced rectal adenocarcinoma Metastatic rectal adenocarcinoma Rectosigmoid adenocarcinoma Recurrent rectal adenocarcinoma Recurrent rectosigmoid carcinoma Stage III rectal cancer AJCC v8 Stage IIIA rectal cancer AJCC v8 Stage IIIB rectal cancer AJCC v8 Stage IIIC rectal cancer AJCC v8 Stage IV rectal cancer AJCC v8 Stage IVA rectal cancer AJCC v8 Stage IVB rectal cancer AJCC v8 Stage IVC rectal cancer AJCC v8	Procedure: biospecimen collection	–
NCT02838836	–	Recruiting	NSCLC Esophageal caner Gastric caner Pancreatic caner Hepatocellular caner CRC	Procedure: study sample collection	–
NCT02813928	–	Completed	CRC	Genetic: ccfDNA analysis	–
NCT02556281	–	Unknown	Colorectal neoplasms	Biological: blood sampling	–
NCT02554448	–	Unknown	Rectal neoplasms CTCs	Other: ISET system	–
NCT01722903	–	Completed	Stage IV CRC Liver metastases Lung metastases	–	–
NCT01671891	–	Unknown	Rectal caner	Radiation: radiation therapy Drug: capecitabine (625 mg/m2, bid, d1‐5 qw) and oxaliplatin (85 mg/m2 d1 qw)	–
NCT01286883	–	TERMINATED	CRC	–	–
NCT01212510	–	Completed	Metastatic CRC	Other: blood sampling	–
NCT01196130	–	Completed	CRC	Behavioral: cancer symptom questionnaire Other: biomarker testing	–

*Note*: –, Not applicable/no results available.

Abbreviations: AJCC, American Joint Commission on Cancer; CC, colon cancer; ccfDNA circulating cell‐free DNA; CRC, colorectal cancer; CTC, circulating tumor cell; EGFR, epidermal growth factor receptor; MRD, minimal residual disease; NSCLC, non‐small cell lung cancer.

CRC is a disease especially amenable to liquid biopsy‐based techniques given the high detachment of circulating tumor fragments (cells, DNA, methylation markers, etc.). Whilst gradual emergence of ctDNA as part of molecular profiling paradigm, tissue biopsy is still general gold standard for solid tumors. In clinical terms, blood ctDNA testing is also less useful in CRC patients suffering from peritoneal carcinomatosis or brain metastases owing to presence of a blood barrier. Innovative strategies for employing ctDNA assays in other body fluids (cerebrospinal fluid, ascites, pleural fluid, etc.) are currently being pursued.^154^

## 5 CONCLUSION AND PERSPECTIVE

Up to now, based on genetic and epigenetic characteristics, classical transcriptional taxonomies, and multi‐omics analysis, numerous studies of CRC molecular subtypes have made great achievements. Moreover, exploration of subtypes through cell lines, animal models, PDXs, organoids, and clinical trials in recent years also contributes to refining biological insights and unraveling subtype‐specific therapies in CRC. On top of the aforementioned advancement, precise treatment strategies that focus on molecular typing of CRC contribute to better stratification of clinical patients, which conduce to clear treatment direction and avoid undertreatment or overtreatment in clinical practice.

However, impact of TME, interference of potential regulatory factors and mechanisms between different subtypes, tumor stroma, and immune components (e.g., fibroblasts and cytotoxic T cells), could influence accurate CRC subtyping, thus impeding clinical practice of CRC. There are also challenges in heterogeneity barriers in CRC. Exhilaratingly, there are latent nascent CRC treatment regimens to reconcile these seemingly paradoxical observations. Therapeutic interventions including nanotechnology, CRISPR/Cas9, organoids, gut microbiome, and liquid biopsy are powerful tools with the possibility to shift the immunologic landscape and outlook for CRC precise medicine.

While our understanding of precise subtypes and cutting‐edge technologies has improved in recent years, our findings highlight substantial gaps in current research that need to be filled before this knowledge can be used to the benefit of patients. We expect that implementation of CRC precise subtypes could create better outgrowths for selection of treatment tactics than current clinical trial standards. Ultimately, this will help move beyond the “one‐size‐fits‐all” regimen and enhance CRC prognosis for more patients. CRC precise subtypes and precise medicine are still in non‐stop exploration.

## AUTHOR CONTRIBUTIONS

**Qin Dang:** Conceptualization (equal); data curation (equal); formal analysis (equal); funding acquisition (equal); investigation (equal); methodology (equal); project administration (equal); resources (equal); software (equal); supervision (equal); validation (equal); visualization (equal); writing – original draft (equal); writing – review and editing (equal). **Lulu Zuo:** Conceptualization (equal); formal analysis (equal); funding acquisition (equal); investigation (equal); methodology (equal); project administration (equal); resources (equal); software (equal); supervision (equal); validation (equal); visualization (equal); writing – original draft (equal); writing – review and editing (equal). **Xinru Hu:** Conceptualization (equal); data curation (equal); funding acquisition (equal); investigation (equal); methodology (equal); project administration (equal); resources (equal); software (equal); supervision (equal); validation (equal); visualization (equal); writing – original draft (equal). **Zhaokai Zhou:** Conceptualization (equal); data curation (equal); formal analysis (equal); funding acquisition (equal); investigation (equal); methodology (equal); project administration (equal); validation (equal); visualization (equal); writing – review and editing (equal). **Shuang Chen:** Conceptualization (equal); data curation (equal); formal analysis (equal); funding acquisition (equal); investigation (equal); methodology (equal); project administration (equal); writing – review and editing (equal). **Shutong Liu:** Conceptualization (equal); data curation (equal); formal analysis (equal); funding acquisition (equal); investigation (equal); methodology (equal); project administration (equal). **Yuhao Ba:** Conceptualization (equal); data curation (equal); formal analysis (equal); funding acquisition (equal); investigation (equal); software (equal); supervision (equal); validation (equal). **Anning Zuo:** Conceptualization (equal); funding acquisition (equal); investigation (equal); methodology (equal); project administration (equal); supervision (equal); validation (equal); visualization (equal). **Hui Xu:** Conceptualization (equal); data curation (equal); formal analysis (equal); funding acquisition (equal); supervision (equal); validation (equal); visualization (equal). **Siyuan Weng:** Conceptualization (equal); data curation (equal); formal analysis (equal); funding acquisition (equal); software (equal); supervision (equal); validation (equal); visualization (equal). **Yuyuan Zhang:** Conceptualization (equal); data curation (equal); formal analysis (equal); funding acquisition (equal); supervision (equal); validation (equal); visualization (equal). **Peng Luo:** Conceptualization (equal); data curation (equal); formal analysis (equal); funding acquisition (equal); investigation (equal); supervision (equal); validation (equal); visualization (equal). **Quan Cheng:** Conceptualization (equal); data curation (equal); formal analysis (equal); supervision (equal); validation (equal); visualization (equal). **Zaoqu Liu:** Resources (equal); software (equal); supervision (equal); validation (equal); visualization (equal). **Xinwei Han:** Resources (equal); software (equal); supervision (equal); validation (equal); visualization (equal).

## FUNDING INFORMATION

This study was supported by Henan Provincial Science and Technology Research Project (Grant No. 221100310100).

## CONFLICT OF INTEREST STATEMENT

The authors declare no potential conflicts of interest.

## Data Availability

Not applicable.
